# The role of N^6^-methyladenosine (m^6^A) modification in the regulation of circRNAs

**DOI:** 10.1186/s12943-020-01224-3

**Published:** 2020-06-10

**Authors:** Lele Zhang, Chaofeng Hou, Chen Chen, Yaxin Guo, Weitang Yuan, Detao Yin, Jinbo Liu, Zhenqiang Sun

**Affiliations:** 1 Department of Colorectal Surgery, The First Affiliated Hospital of Zhengzhou University, Zhengzhou, 450052 Henan China; 2 Department of Colorectal Surgery, Zhengzhou Central Hospital, Zhengzhou University, Zhengzhou, 450007 Henan China; 3 Department of Thyroid Surgery, The First Affiliated Hospital of Zhengzhou University, Zhengzhou, 450052 Henan China; 4 Academy of Medical Sciences, Zhengzhou University, Zhengzhou, 450052 Henan China; 5 School of Life Sciences, Zhengzhou University, Zhengzhou, 450001 Henan China; 6 School of Basic Medical Sciences, Zhengzhou University, Zhengzhou, 450002 Henan China

**Keywords:** M^6^A, CircRNA, M^6^A modified circRNA, Innate immunity, Tumour

## Abstract

N^6^-methyladenosine (m^6^A), the most abundant modification in eukaryotic cells, regulates RNA transcription, processing, splicing, degradation, and translation. Circular RNA (circRNA) is a class of covalently closed RNA molecules characterized by universality, diversity, stability and conservatism of evolution. Accumulating evidence shows that both m^6^A modification and circRNAs participate in the pathogenesis of multiple diseases, such as cancers, neurological diseases, autoimmune diseases, and infertility. Recently, m^6^A modification has been identified for its enrichment and vital biological functions in regulating circRNAs. In this review, we summarize the role of m^6^A modification in the regulation and function of circRNAs. Moreover, we discuss the potential applications and possible future directions in the field.

## Background

Circular RNA (circRNA) is a class of single-stranded covalently closed RNA molecules that was first discovered in pathogens by Sanger et al. in 1976 [1]. It is now generally accepted that circRNA is generated by a process named back-splicing [2], and increasing studies have demonstrated that circRNA plays important roles in the occurrence, development and prognosis of various diseases, including tumorigenesis [3–5], neurodevelopmental processes [6] autoimmune responses [7], and infertility [8]. However, studies on how circRNA is regulated before exerting specific biological functions are still limited [9].

To date, over 160 types of chemical modifications have been identified in RNA molecules, of which methylation is the most common type [10]. The methods of methylation modifications of RNA include N^6^-methyladenosine (m^6^A), 5-methylcytosine (m^5^C), N1-methyladenosine (m^1^A), 5-hydroxymethylcytosine (5hmC), N6, 2′-O-dimethyladenosine (m^6^Am), 7-methylguanine (m^7^G), etc. [11], of which m^6^A modification is the most abundant type in eukaryotic cells [12]. Previous studies have shown that m^6^A modification is a dynamic and reversible process and regulates RNA transcription, processing, splicing, degradation, and translation [13–17]. The occurrence and development of many diseases, such as tumours [18], obesity [19], infertility [20], autoimmune disease [21] and neurological disease [22], are closely related to alteration of m^6^A modification.

Although research on the regulatory mechanism of m^6^A modification of mRNA has made great progress [23], for some non-coding RNAs, especially circRNAs, the regulatory network of m^6^A has not been fully elucidated [24]. In this review, we summarize the role of m^6^A modification in circRNA regulation and function. Furthermore, we discuss the potential applications and possible future directions in this field.

### M^6^A writers, erasers, and readers

The regulation function of m^6^A is mainly accomplished by three homologous factors referred to as “writers”, “erasers” and “readers”. M^6^A “writers” are proteins involved in the formation of the methyltransferase complex, including methyltransferase-like 3 and 14 proteins (METTL3 and METTL14) and their cofactors WT1 associated protein (WTAP), RNA-binding motif protein 15/15B (RBM15/15B), Vir-like m^6^A methyltransferase associated (VIRMA), and zinc finger CCCH-type containing 13 (ZC3H13); METTL3, as the earliest identified and most well-known component [25], is an S-adenosylmethionine (SAM) binding protein and is highly conserved in various eukaryotic species [26, 27]. Notably, except for the above readers that function in a form of complexes, a homologue of METTL3 (METTL16) has been identified as a novel independent RNA methyltransferase that regulates cellular SAM levels and methylates U6 small nuclear RNA [28].

**Fig. 1:**
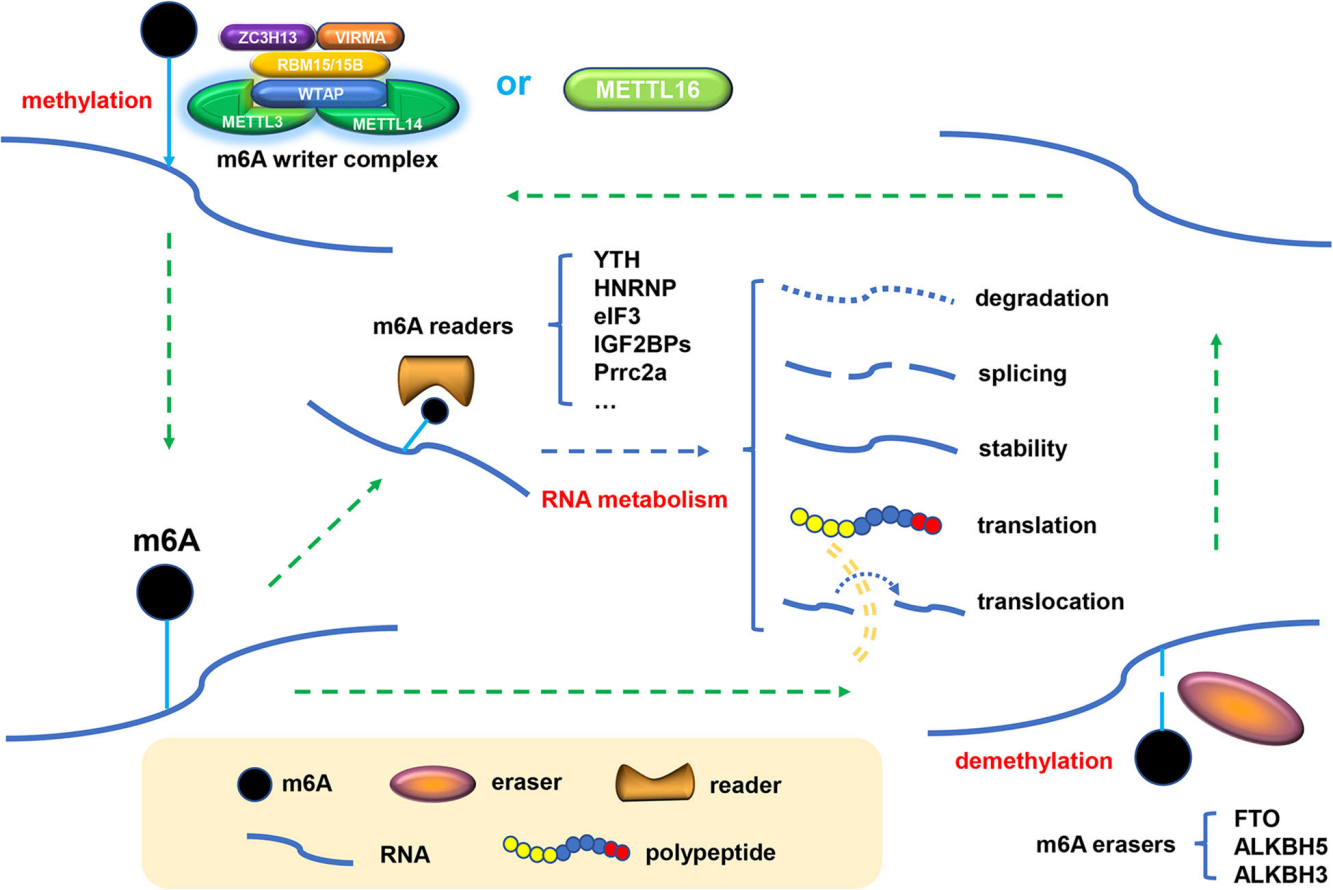
Dynamic and reversible m^6^A process. The installation, removal and identification of m^6^A are conducted by writers, readers, and erasers, respectively. Writers refer to the m^6^A complex, including METTL3, METTL14, WTAP, RBM15/15B, VIRMA and ZC3H13. Besides, METTL16 is a novel independent RNA methyltransferase. Erasers are proteins that own demethylases activity, including FTO, ALKBH5, ALKBH3. Readers are proteins that recognize the m^6^A modification and perform multiple functions in RNA metabolism, some of which identified so far are YTH family, HNRNP family, eIF3, IGF2BPs and Prrc2a

The dynamic and reversible m^6^A process (Fig. 1) also relies on some demethylases (erasers). Fat mass and obesity-associated protein (FTO), the first protein identified to catalyse m^6^A demethylation [29], works together with a homologue of itself (ALKBH5, [30] to maintain the balance of m^6^A levels in the transcriptome [31]. ALKBH3 is a recently discovered demethylase that prefers to perform its demethylation function on tRNA rather than on mRNA or rRNA [32]. In addition, ALKBH3 is also a generally accepted DNA repair enzyme and has the potential to be a molecular marker for tumours [33]. M^6^A-modified RNA requires a class of variable RNA-binding proteins (readers) to perform specific biological functions. Proteins of the YT521-B homology (YTH) domain family, including YTHDC1, YTHDC2, YTHDF1, YTHDF2 and YTHDF3 [34], were the first five characterized m^6^A readers in humans that have a conserved m^6^A-binding domain. The heterogeneous nuclear ribonucleoprotein (HNRNP) family is another group of RNA-binding proteins (RBPs) that serves as m^6^A readers. Heterogeneous nuclear ribonucleoprotein A2/B1 (HNRNPA2B1) specifically recognizes m^6^A-modified RNA and acts as a mediator in m^6^A-dependent nuclear RNA processing [35]. In contrast, HNRNPC and HNRNPG cannot directly bind to the m^6^A site, but they can mediate the selective splicing process of transcripts containing m^6^A modification by identifying and binding to the m^6^A-dependent structural switches [36]. Translation initiation factor 3 (eIF3) initiates the translation procedure by binding to the m^6^A site in the 5′-UTR of mRNA, while the family of insulin-like growth factor 2 mRNA-binding proteins (IGF2BPs, including IGF2BP1/2/3) makes the target gene and the corresponding translation more stable [37]. Moreover, proline rich coiled-coil 2 A (Prrc2a) is a novel m^6^A reader that stabilizes mRNA expression by binding to a consensus GGACU motif in the coding sequence (CDS) in an m^6^A-dependent manner [38].

The dynamic reversibility of m^6^A modification is closely associated with the normal physiological activities of the organism. Studies have revealed that m^6^A-modified mRNA or non-coding RNA (mainly miRNA and lncRNA) plays crucial roles in spermatogenesis [39], T cell homeostasis [40], Drosophila sex determination [41], heat shock responses [42], reprogramming and pluripotency [43], as well as other processes. Considering the significance of m^6^A modification in the regulation of gene expression and various biological functions, dysregulation of m^6^A levels contributes to diverse diseases, especially for some cancers. Recent studies have indicated that both aberrant m^6^A modification and abnormal expression of m^6^A regulatory proteins can both be detected in acute myeloid leukaemia (AML) [44], hepatocellular carcinoma (HCC) [45], glioblastoma stem cells (GSCs) [46], breast cancer [47], obesity [19], infertility [20], autoimmune disease [21] and neurological disease [22].

### Characteristics, regulatory mechanisms and biological functions of circRNA

According to their origin, circRNAs can be classified into four broad categories, exonic circRNAs (ecircRNAs), intronic circRNAs (ciRNAs), exon-intron circRNAs (EIciRNAs) and others, ranging from virus, tRNA, rRNA, snRNA [48]. In general, circRNAs can be detected in most organisms, including archaea [49], plants [50], parasites [51], and most mammals [52]. Previous studies have shown that there are more than 25,000 different RNAs that generate corresponding circRNAs in human fibroblasts [53]. Different circRNAs can also be produced by the same gene through alternative circularization [54], which causes the diversity of circRNAs. Another important characteristic of circRNAs is that they cannot be degraded by exonucleases and are therefore more stable than linear circRNAs [55]. Homology studies between different species have shown that circRNAs are highly conserved in evolution between species. The level of homology of circRNA in mice and humans reaches 20% or more [56], while that in pigs and mice is between 15 and 20% [57]. The last but most practical characteristic of circRNAs is that their expression levels vary according to different tissues and different growth stages, which is an essential characteristic for an ideal disease biomarker. Expression profiles of different tissues in humans and mice show that nerve tissue (especially brain tissue) contains more circRNA than other tissues [58], and the expression level of circRNA is gradually upregulated with the development of the brain.

Based on adequate studies on the characteristics of circRNA, an increasing number of studies have focused on its regulatory function [59, 60] (Table 1). The most classical network in which circRNA exerts a specific function occurs through acting as competing endogenous RNA (ceRNA). CircRNAs with a miRNA response element (MRE) can bind specific miRNAs to negatively regulate their activity, so circRNAs can also be considered “miRNA sponges”. The first circRNA defined as an “miRNA sponge” was ciRS-7, and it was first identified in human and mouse brains by Thomas B et al. in 2013 [72]. In addition, circRNAs can also perform specific physiological functions by interacting with some RBPs. In most cases, these circRNAs act as a “separant” to inhibit the function or transport of RBPs. CircEIF3J and circPIAP2, which are predominantly detected in the nucleus, can interact with U1 snRNP and promote transcription of their parental genes [73]. Interestingly, some circRNAs located in the cytoplasm have similar protein binding abilities. CircFoxo3 interacts with inhibitor of DNA binding 1 (ID-1), E2F transcription factor 1 (E2F1), focal adhesion kinase (FAK), and hypoxia inducible factor 1 subunit α (HIF1-α) so that these components are retained together in the cytoplasm [74]. Moreover, recent studies have shown that some circRNAs could be translated into proteins [75, 76]. In the absence of a dissociative 5′ end, the translation of circRNAs cannot be initiated by traditional cap-dependent regulatory elements and therefore requires an internal ribosome entry site (IRES) or other elements to activate a cap-independent pathway. To support this claim, Wang et al. engineered an IRES in a circRNA and then corresponding protein translated by this circRNA was detected in 293 T cells [77]. Recently, another study found that m^6^A modification was abundant in many circRNAs, and this kind of methylation modification could drive circRNA translation in a manner similar to IRES [78].

**Table 1:** Roles of circRNA in different cancers

Functions	CircRNA	Cancer	Dysregulation	References
MiRNA sponge	circ_0026134	Lung cancer	Up	[61]
	circ_0005963	Colorectal cancer	Up	[62]
	circ_000684	Gastric cancer	Up	[63]
	circ_0051443	Hepatocellular cancer	Down	[64]
Binding to protein	circ-Amotl1	Breast cancer	Up	[65]
	circ-Foxo3	Breast cancer	Down	[66]
	circ-ZKSCAN1	Hepatocellular cancer	Down	[67]
Translation template	circ-FBXW7	Glioblastoma	Down	[68]
	circ-SHPRH	Glioblastoma	Down	[69]
	circ-PPP1R12A	Colon cancer	Up	[70]
	circ-β-catenin	Liver cancer	Up	[71]

**Fig. 2:**
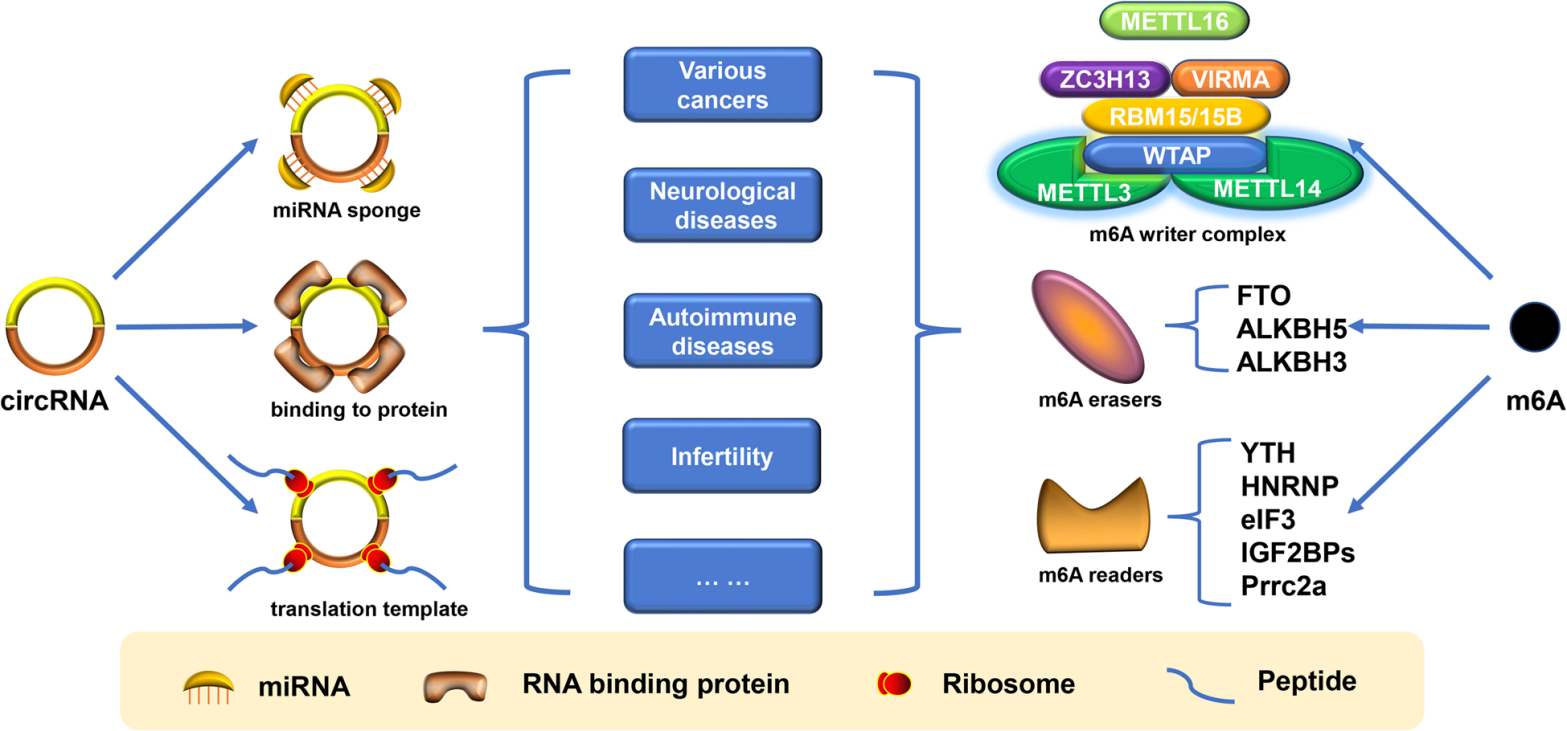
Role of circRNA and m^6^A modification in various diseases. Three major biological functions of circRNAs are shown on the left. Three homologous factors involved in the regulatory function of m^6^A are listed on the right

Although still in its infancy, circRNAs have been found to be closely related to the occurrence, development and prognosis of various diseases (Fig. 2). Recent studies have demonstrated that the dysregulation of circRNAs exists in different cancers, neuropsychological diseases, autoimmune diseases, infertility, diabetes, nephropathy, arthritis, etc., but few of these circRNAs have been verified to have biological functions. Some studies considered that it might be related to the epigenetic modification of circRNA [79, 80], and m^6^A modification is the first role that comes into sight.

### Role of m^6^A methylation in the regulation of circRNAs

Current studies have identified that dysregulation of m^6^A modification contributes to various diseases, especially for some cancers. Generally, m^6^A functions as a double-edged sword. In most cases, aberrant m^6^A modification contributes to tumorigenesis and tumour progression. However, recent studies revealed that abnormal m^6^A level can also cause tumour suppression [81]. Since m^6^A functions via affecting RNA metabolism primarily, researchers have focused their attention on m^6^A-modified mRNA in recent years. Currently, m^6^A-modified ncRNAs, especially m^6^A-modified circRNAs, remain to be further explored. Here, we summarize the role of m^6^A modification in circRNA regulation and function.

#### M^6^A modification regulates circRNA translation

**Fig. 3:**
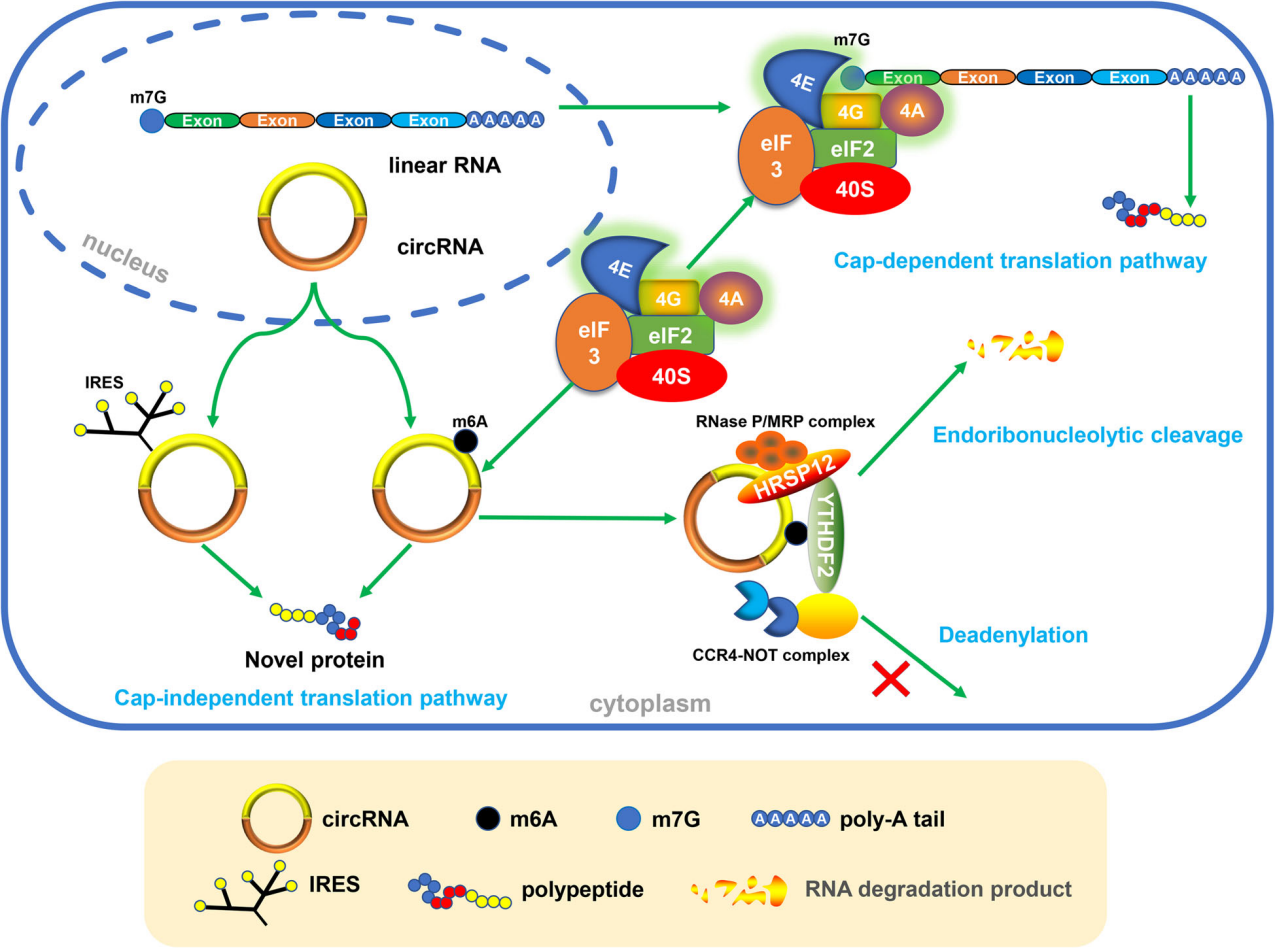
M^6^A modification regulates circRNA translation and degradation. The translation of circRNAs requires m^6^A modification or IRES, which is different from the traditional cap-dependent pathway of linear RNAs. M^6^A-modified circRNAs are endoribonuclease-cleaved via the YTHDF2-HRSP12-RNase P/MRP axis

Recent studies have shown that some circRNAs have protein-coding potential [75, 82], and the translation process can be driven by m^6^A [78]. In general, the translation of RNA in eukaryotic cells requires a eukaryotic translation initiation factor 4F (eIF4F) complex, which is composed of three initiation factors, eIF4A (a helicase protein), eIF4E (a m^7^G reader) and eIF4G (a scaffold protein) [83]. On mRNA, these transcription initiation elements are located on the cap structure of the 5′ end, so here we define it as a cap-dependent pathway [84]. However, this traditional cap-dependent pathway does not work in a closed circular transcript in the absence of a dissociative 5′ end. Therefore, some cap-independent translation initiation mechanisms, such as the IRES-dependent pathway and m^6^A-dependent pathway (Fig. 3), have been proposed to explain the protein-coding ability of some circRNAs. IRESs are sequences that mediate the binding between ribosomes and RNA, thus initiating translation. The reported protein-coding circRNAs driven by IRES include circZNF609 in myogenesis [75], circMbL in fly head extracts [82], circSHPRH and circFBXW7 in glioma tumorigenesis [68, 69], and circβ-catenin in liver cancer growth [71].

However, a recent study conducted by Yang et al. broadens our horizons on the coding landscape of the human transcriptome. An m^6^A-driven translation pathway was proposed and verified in cellular responses to environmental stress [78]. In this study, circRNAs containing m^6^A motifs were detected to be translated, and the efficiency of translation was validated to be modulated by the m^6^A level. Mechanistically, this m^6^A-driven translation was initiated by factor eIF4G2 and m^6^A reader YTHDF3, enhanced by methyltransferase METTL3/14, and inhibited by demethylase FTO. Moreover, the m^6^A level of some endogenous circRNAs was tested, and the results showed that the m^6^A motif was abundant in circRNAs. In terms of the whole human transcriptome, m^6^A-modified circRNAs with coding potential are not rare [85, 86]. Finally, 33 endogenous peptides encoded by the back-splice junctions of circRNAs were chosen for functional analysis. However, regrettably, no functional enrichment was detected despite the translation of these circRNAs being indeed elevated when facing cellular stress.

Notably, these two cap-independent translation pathways might not function independently. Legnini et al. reanalysed m^6^A-Seq and immunoprecipitation data [15] and combined the data with other m^6^A immunoprecipitation (IP) results in myoblasts alone [75]. The results showed that a high m^6^A methylation level was detected in the IRES-activated protein-coding circRNA circZNF609, suggesting a possible connection between these two cap-independent pathways.

#### M^6^A modification facilitates circRNA degradation

Due to their closed circular structure, circRNAs are naturally more stable than their parental linear RNAs, as they are not the primary targets of foreign chemicals or exonucleases. This has been validated by many studies related to the characterization of circRNAs [59, 79]. CircRNAs are rarely degraded prior to the corresponding parental linear circRNAs in Actinomycin D and RNase R treatment. However, how circRNA is degraded and what factors contribute to the surveillance pathway remain largely unknown.

A previous study reported that circRNAs with near perfect complementary miRNA target sites could be degraded in an Ago2-slicer-dependent manner, but for those circRNAs without miRNA sponge function or specific microRNA target sites, this method does not work [87]. Another study found that the depletion of GW182 (a key component of the P-body and RNAi machine) resulted in the accumulation of endogenous circular transcripts. However, the depletion of other P-body components or RNAi complex factors did not have similar effects, indicating that GW182, not the P-body or RNAi machine, affected the degradation of circRNAs [88]. Regrettably, GW182 shows little effect on the nuclear export of circRNAs, and its functions in the cytoplasm has not been fully elucidated, so other studies are needed to explain the degradation of circRNA.

The endoribonucleolytic cleavage pathway is one of the pathways by which m^6^A-modified RNAs are degraded. As a new star in the field of non-coding RNA research, m^6^A-modified circRNAs were also found to be endoribonuclease-cleaved via a YTHDF2-HRSP12-RNase P/MRP axis [89] (Fig. 3). HRSP12 is an adaptor protein that bridges YTHDF2 (m^6^A reader protein) and RNase P/MRP (endoribonucleases) to form a YTHDF2-HRSP12-RNase P/MRP complex, for which YTHDF2 is the guide. When an m^6^A-modified circRNA is recognized by YTHDF2, regardless of whether it occupies an HRSP12-binding site, RNase P/MRP always performs its endonuclease function. The only difference is that the existence of the HRSP12 binding site greatly improves the efficiency of endoribonucleolytic cleavage. Subsequently, the m^6^A-modified circRNA is selectively downregulated. What follows is a change in the biological function of circRNAs. Thus, we can conclude that one of the ways that m^6^A modification regulates the biological function of circRNAs is to affect their degradation.

#### M^6^A modified circRNA in innate immunity

Innate immunity (also named non-specific immunity) is the natural immune defence function formed by the body in the process of development and evolution. It plays a decisive role in controlling and resolving the inflammatory response to tissue damage [90]. A recent study found that innate immunity can be activated differently by exogenous and endogenous RNAs [91].

**Fig. 4:**
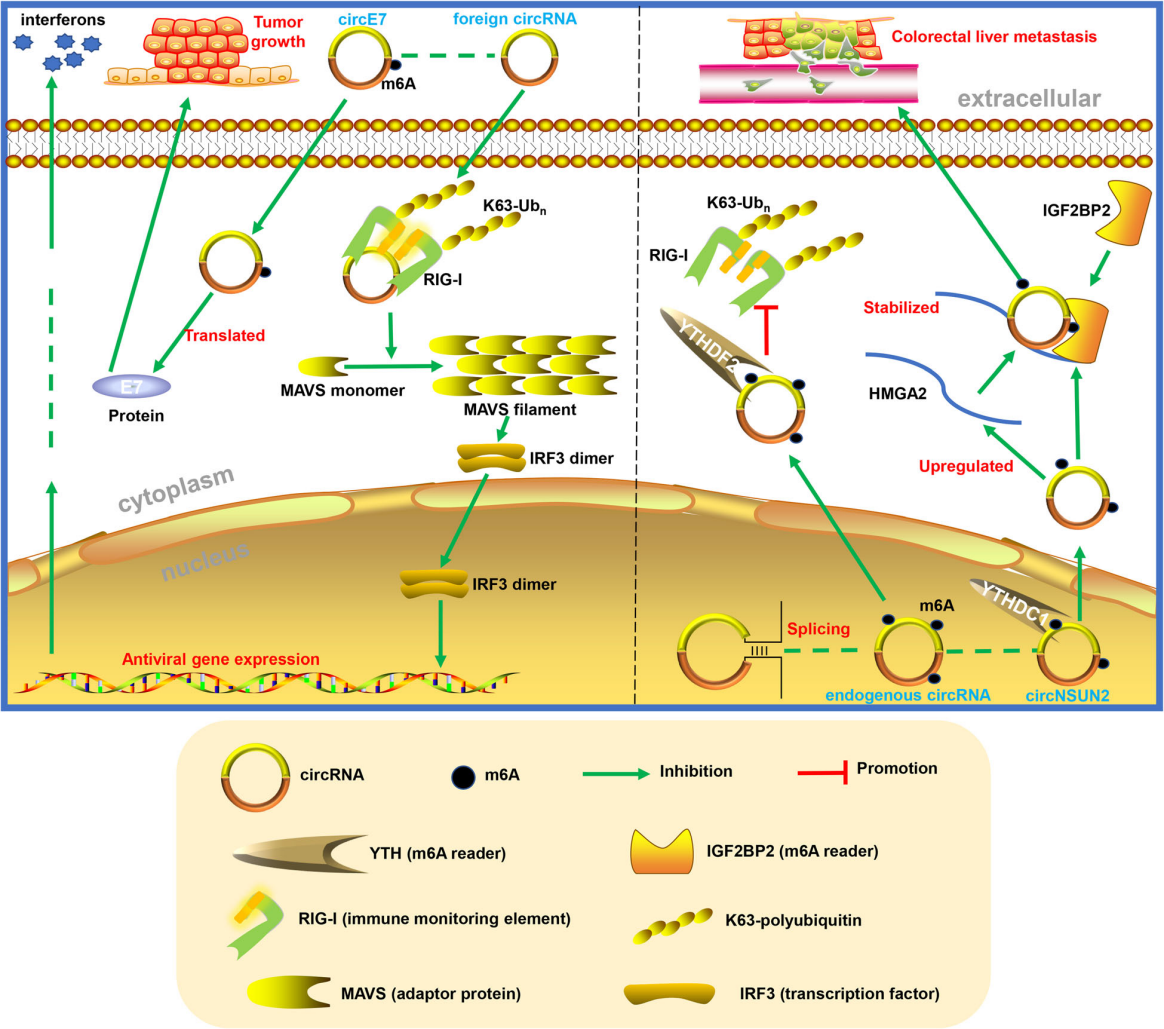
M^6^A-modified circRNAs in innate immunity and tumours. M^6^A modification defines endogenous versus foreign circRNA in innate immunity. M^6^A modification of circNSUN2 promotes the liver metastasis of colorectal cancer by facilitating cytoplasmic export and forming a circNSUN2/IGF2BP2/HMGA2 RNA-protein ternary complex to stabilize HMGA2 mRNA

All transcripts directly generated by RNA polymerase II bear an m^7^G cap, and RIG-I (also known as DDX58) senses a triphosphate at the 5′ end [92]; these are essential elements for immune monitoring. Due to the closed circular structure, circRNAs are supposed to be able to escape from the end monitoring system. However, recent studies showed that the invasion of some exogenous circRNAs still leads to potent induction of innate immunity genes and confers protection against viral infection [93], while endogenous circRNAs form some 16–26 bp imperfect RNA duplexes to resist the double-stranded RNA (dsRNA)-activated protein kinase (PKR) in innate immunity [94] (Fig. 4). One of the explanations was found to describe how the immune system defined endogenous versus foreign circRNA as m^6^A modification.

A study conducted by Y. Grace et al. found that a circRNA generated by ZKSCAN1 introns (circSELF), but not autocatalytic splicing (circFOREIGN), is associated with WTAP and KIAA1429 (m^6^A writers) as well as YTHDF2 and HNRNPC (m^6^A readers) [80]. Further research found that different levels of m^6^A modification were detected in these two circRNAs, and m^6^A modification marked circRNA as “SELF”. CircSELF can escape innate immunological surveillance via YTHDF2-mediated suppression, which is consistent with a recent study showing that m^6^A-modified RNAs could be recruited by YTHDF proteins and induced into phase-separated condensates via their N-terminal disordered domains [95]. These results suggest that human circRNAs may be marked by the covalent m^6^A modification, which is essential for the recognition function of innate immunity.

#### M^6^A-modified circRNA in tumours

Since m^6^A and circRNAs are both closely related to tumours, it is natural to speculate that m^6^A modification might regulate the function of circRNAs in various tumours. Herein, we briefly review recent studies of m^6^A-modified circRNAs associated with tumours.

As the third most prevalent and the second most deadly malignancy worldwide, colorectal cancer is still a major threat to human health, especially in China [96]. Clinically, the liver metastasis of colorectal cancer is the most common organ metastasis and leads to poor prognosis beyond 5 years [97]. Recently, Chen et al. found that m^6^A modification of circNSUN2 promotes the liver metastasis of colorectal cancer by facilitating cytoplasmic export and forming a circNSUN2/IGF2BP2/HMGA2 RNA-protein ternary complex to stabilize HMGA2 mRNA [79] (Fig. 4). HMGA2, a high mobility group AT-hook 2, is already widely believed to be related to the progression of colorectal cancer [98, 99]. These results illuminate how m^6^A modification affects the interaction between circRNA and RBP.

Cervical cancer is a prevalent gynaecological cancer with a relatively poor prognosis [100], and almost all cervical cancers are caused by oncogenic types of human papillomavirus (HPV) [101]. CircE7 is an oncoprotein-encoding circRNA generated by HPV that is closely related to the growth of CaSki cervical carcinoma cells both in vitro and in vivo. Interestingly, m^6^A modification is detected and verified to be an essential motif for the protein-coding ability of circE7 [102], which is consistent with the ideas mentioned above that m^6^A modification facilitates circRNA translation and helps foreign circRNAs escape immune monitoring. Moreover, circE7 is not a special case that is specifically expressed or modified by m^6^A. Another study identified more than 1 thousand m^6^A-modified circRNAs in human embryonic stem cells (hESCs) and showed that m^6^A circRNAs are also abundant in HeLa cells [103], which expands our understanding of the breadth and regulatory aspects of m^6^A modification.

In addition to modifying circRNA directly, m^6^A can also affect the function of circRNA via changing the methylation state of downstream molecules. As one of the main response factors downstream of the Hippo pathway, YAP is closely related to the occurrence and development of various tumours [104, 105]. In hepatocellular cancer, circ_104075 can absorb miR-582-3p to stimulate tumorigenesis via YAP [106]. M^6^A modification in the 3′-UTR of YAP induces the interaction with miR-382-5p and subsequently leads to the inhibition of YAP. Then, the promoting effect of circRNA_104075 on hepatocellular cancer is inhibited. In addition, a combinative bioinformatics prediction of m^6^A level, IRES and open reading frame (ORF) could indicate the protein-coding potential of circPVRL3 in gastric cancer [107].

### Applications and future directions

Considering the stability and conserved nature of their structure, the potential of circRNAs as diagnostic biomarkers and therapeutic targets is unquestionable and is supported by the growing number of circRNA-related studies in recent years [108]. However, the relationship between epigenetic modification and circRNA functions is still largely unknown. As one of the most abundant RNA modifications, m^6^A provides us with an intermediate mechanism by which circRNAs are regulated by upstream molecules and allows us to predict and interfere with disease progression caused by the dysregulation of circRNAs. There is no doubt that it would greatly expand our understanding of circRNA and drive its applications.

Notably, no specific biological functions have been detected in the majority of already discovered circRNAs, which is also one of the reasons that circRNAs were regarded as by-products of splicing when first discovered [109]. Considering the ubiquitous m^6^A modification in annotated functional circRNAs, we speculate that it might be related to the tissue and developmental stage specificity of circRNA. That is, specific circRNAs present differential expression only if they have been activated by specific molecular mechanisms, such as m^6^A, in specific tissues, developmental stages and subcellular locations. To test this conjecture, a combination analysis of the m^6^A Hi-Res chip and RNA-seq would be helpful for our future research on the biological function and clinical application of m^6^A-modified circRNAs.

## Conclusions

With the broad application of high-throughput sequencing technology and bioinformatics analysis in scientific research, increasing numbers of m^6^A-modified circRNAs will be found and tested. By then, our understanding of how m^6^A modification regulates circRNA will not be confined to the four limited aspects of translation, degradation, immunity, and tumours. Other effects of m^6^A on circRNA, such as processing or splicing effects, and the biological functions of m^6^A-modified circRNAs in other non-neoplastic diseases could be further investigated.

Since the current understanding of m^6^A-modified circRNAs is only at the tip of the iceberg, there is still a long way to go to reveal its further regulatory mechanisms and subsequent biological functions in diseases. At this stage, we propose that more m^6^A regulated circRNAs could be developed to diagnostic biomarkers and therapeutic targets in the future. With the existing technical advancements, it is no longer a technical problem to identify the characterization, localization, transport and degradation of circRNAs in living cells. We anticipate that methods for simplifying the detection of m^6^A levels of specific circRNAs and for effectively extracting circRNAs with low abundance in limited samples, such as exosomal circRNAs, will progress in the field.

## Data Availability

Not applicable.

## Abbreviations

5hmC: 5-hydroxymethylcytosine
AML: Acute myeloid leukaemia
CDS: Coding sequence
ceRNA: Competing endogenous RNA
CircRNA: Circular RNA
ciRNAs: Intronic circRNAs
E2F1: E2F transcription factor 1
ecircRNAs: Exonic circRNAs
EIciRNAs: Exon-intron circRNAs
eIF3: Translation initiation factor 3
FAK: Focal adhesion kinase
FTO: Fat mass and obesity-associated protein
GSCs: Glioblastoma stem cells
HCC: Hepatocellular carcinoma
hESCs: Human embryonic stem cells
HIF1-α: Hypoxia inducible factor 1 subunit α
HNRNP: Heterogeneous nuclear ribonucleoprotein
HPV: Human papillomavirus
ID-1: Inhibitor of DNA binding 1
IGF2BPs: Insulin-like growth factor 2 mRNA-binding proteins
IP: Immunoprecipitation
IRES: Internal ribosome entry site
M^1^A: N1-methyladenosine
M^5^C: 5-methylcytosine
M^6^A: N^6^-methyladenosine
M^6^Am: N6, 2′-O-dimethyladenosine
M^7^G: 7-methylguanine
METTL14: Methyltransferase-like 14 protein
METTL3: Methyltransferase-like 3 protein
MRE: miRNA response element
ORF: Open reading frame
PRCC2A: Proline rich coiled-coil 2 A
RBM15/15B: RNA-binding motif protein 15/15B
RBPs: RNA-binding proteins
SAM: S-adenosylmethionine
VIRMA: Vir-like m^6^A methyltransferase associated
WTAP: WT1 associated protein
YTH: YT521-B homology
ZC3H13: Zinc finger CCCH-type containing 13
